# Whole mitochondrial genomes reveal the relatedness of the browsing ant incursions in Australia

**DOI:** 10.1038/s41598-023-37425-1

**Published:** 2023-06-24

**Authors:** M. Asaduzzaman Prodhan, Marc Widmer, Tonny Kinene, Monica Kehoe

**Affiliations:** DPIRD Diagnostics and Laboratory Services, Department of Primary Industries and Regional Development, 3 Baron-Hay Court, South Perth, WA 6151 Australia

**Keywords:** Genomics, Next-generation sequencing, Entomology

## Abstract

Global trade and human movements outspread animal species, for example ants, from their native habitats to new areas. This causes biosecurity concerns because an exotic ant might have adverse impacts on agriculture, the environment, or health; thus, incurring economic losses. The browsing ant, *Lepisiota frauenfeldi*, was first detected in 2013 at the Perth Airport. Since then, more discrete browsing ant infestations have been found in Perth and at the Ports of Darwin and Brisbane. This exotic ant has been deemed a significant pest in Australia and eradication efforts are underway. However, tackling this invasion requires an understanding of how these infestations are related. Are they same or separate or a combination of both? Here, we carried out a phylogenetic analysis using high-throughput sequencing data to determine their relatedness. Our results showed that each interstate incursion was separate. Furthermore, the Western Australian incursions might have two introductions. These findings are critical in devising effective biosecurity measures. However, we discovered that this information could only be revealed by analysing the whole mitochondrial genome; not by a single mitochondrial gene as typically done for species identification. Here, we sequenced 51 whole mitogenomes including three of its congener *L. incisa* for the first time, for tracing future infestations.

## Introduction

Global trade and human movements are influencing the geographic distribution of animal species^1–4^. Ants are the most studied terrestrial group for dispersal to new geographic locations^2,5^. When an ant species is introduced to a new area, it is often called an exotic or tramp ant^6^. An exotic or tramp ant is categorised as invasive^7,8^ when it spreads in the newly introduced site, dominates the local fauna, and exerts a negative impact on the surrounding ecosystem, biodiversity, environment, or public health^2,9^.

An invasive ant may cause significant economic losses to agriculture^10–15^, livestock^16^, and household properties^2,17^. For example, red imported fire ant (RIFA, *Solenopsis invicta*), a native of South America, caused an estimated US$5–6 billion losses in the USA^2,17,18^. The potential cost impacts in Australia would be in excess of $1.65 billion per year if RIFA were to establish permanently in Australia^2,19^. Furthermore, it adversely affects biota^20,21^, human health^22–25^, and wildlife^26,27^. These economic losses and damages warrant biosecurity measures put in place for early detection and eradication of any exotic pests^28^.

*Lepisiota frauenfeldi*, commonly known as browsing ant, was detected in April 2013 at the Perth Airport precinct, Western Australia. This was the first post border occurrence of the genus *Lepisiota* in Australia^29^. Since then, a further nine discrete browsing ant infestations have been found within the Perth metropolitan area, seven of which are believed to be linked to the original airport infestation. In June 2015 browsing ant was discovered at the Port of Darwin, Northern Territory and then in April 2019 at the Port of Brisbane, Queensland^29^. During surveillance activities for *L. frauenfeldi*, *L. incisia* has also been detected in 14 different locations in Western Australia since 2020, and 10 since 2022.

Browsing ant has been deemed a nationally significant pest and would likely become a considerable horticultural, environmental, and domestic pest if established in Australia. These aggressive ants are native to the Palearctic Bioregion^30^, and are ideally suited to Australian conditions. They form multi-queened super-colonies and monopolise food resources, quickly reaching high populations and displacing other invertebrates^31^.

Through tracing activities, authorities have uncovered 34 browsing ant infestations across Australia since 2013; 10 in Western Australia, 23 in the Northern Territory and one in Queensland^29^. These are all either eradicated or are under eradication, at a cost of many millions of dollars. This demonstrates browsing ants’ genuine capacity for spread and raises the question why they have suddenly emerged as such a prominent biosecurity risk across Australia, in such a relatively short period^31^.

When browsing ant was first discovered in Perth in 2013, very little in the way of written material or scientific literature was available to elucidate their biology, phenology, and pest characteristics such as population dynamics and dispersal mechanisms. And with so many infestations manifesting across Australia over just 6 years, we needed to understand the relatedness of the separate infestations to know if the incursions spread from one original incursion, were separate incursions, or a combination of both. DNA profiling using genetic markers such as ‘Sequence Tandem Repeats (STR) or microsatellites’^32,33^ could answer these questions by revealing any relatedness and even the origin of separate infestations, and also allow for advances in taxonomy and species identification^31^. However, there are currently no publicly available genetic markers or STR for browsing ants. Developing such markers from scratch would be time-consuming^32^ and delay the urgent biosecurity responses required to tackle this invasive pest incursion.

Instead, the relatedness of the incursions can be revealed by inferring the phylogeny of the ants using their mitochondrial genes^34^. Mitochondrial genes have widely been used to infer phylogeny in insects^35,36^. This is because mitochondrial genes are (i) high in copy number making them easier to amplify through the Polymerase Chain Reaction (PCR) assays^37^, (ii) maternally inherited^38^ thus lacking recombination^39^ presumably resulting in similar genealogical history for the entire molecule, (iii) high rate of nucleotide substitution allowing a chance to capture phylogeny signal without intensive sequencing^40^, and (iv) relatively conserved in size, content and synteny in animals^41,42^.

However, recent studies show that the whole mitochondrial genome can reveal a deeper-level phylogeny among the taxa than a single mitochondrial gene^41,43,44^. Furthermore, the recent advancement of sequencing technologies and decrease in sequencing costs make it cost and time effective to sequence the whole mitochondrial genome rather than partial, single or multiple genes^41^. Here, we sequence the whole mitochondrial genomes of 48 *Lepisiota frauenfeldi* individuals representing 10 incursions in Australia and four from overseas. We include *Lepisiota incisa* as an outgroup. Currently, there is no whole mitogenome data for *L. incisa* in Genbank^45^. We have constructed phylogenetic trees using the whole mitochondrial genomes as well as individual protein coding genes to reveal the relatedness of the *L. frauenfeldi* incursions in Australia. At the time of writing, all known infestations of *L. frauenfeldi* and *L. incisa* are either eradicated or under eradication.

## Results

### Phylogenetic analyses

Fifty-one complete mitochondrial genomes (mitogenomes) were sequenced using Oxford Nanopore Technologies (Table 1). Sequencing depth varied from 8 to 1785 × with a median value of 166 × (Table 2). The reconstructed mitogenomes were 16,455–17,430 bases in length. The average value for both N50 and NG50 was 17,116 bases. L50 and LG50 were 1 for all the constructed mitogenomes (Table 2). The reconstructed mitogenomes were subjected to Bayesian Inference and Maximum Likelihood based phylogenetic analysis.

**Table 1:** Browsing ant collection details.

Sample ID	Scientific name	Life stage	Collection location	Collected by	Collection date
B1_5	*Lepisiota frauenfeldi*	Adult	Belmont, WA, Australia	Marc Widmer	2014
B2_4	*Lepisiota frauenfeldi*	Adult	Belmont, WA, Australia	Marc Widmer	2014
B2_5	*Lepisiota frauenfeldi*	Adult	Belmont, WA, Australia	Marc Widmer	2014
Br1_2	*Lepisiota frauenfeldi*	Adult	Brisbane, QLD, Australia	Marc Widmer	2019
Br1_4	*Lepisiota frauenfeldi*	Adult	Brisbane, QLD, Australia	Marc Widmer	2019
Br1_7	*Lepisiota frauenfeldi*	Adult	Brisbane, QLD, Australia	Marc Widmer	2019
Br2_1	*Lepisiota frauenfeldi*	Larva	Brisbane, QLD, Australia	Marc Widmer	2019
Br2_4	*Lepisiota frauenfeldi*	Larva	Brisbane, QLD, Australia	Marc Widmer	2019
PA1_6	*Lepisiota frauenfeldi*	Adult	Perth Airport, WA, Australia	Marc Widmer	2013
PA1_9	*Lepisiota frauenfeldi*	Adult	Perth Airport, WA, Australia	Marc Widmer	2013
PA2_3	*Lepisiota frauenfeldi*	Adult	Perth Airport, WA, Australia	Marc Widmer	2013
PA2_5	*Lepisiota frauenfeldi*	Adult	Perth Airport, WA, Australia	Marc Widmer	2013
TL1	*Lepisiota frauenfeldi*	Adult	Timor Leste	C Brumley—DPIRD	2018
TL2	*Lepisiota frauenfeldi*	Adult	Timor Leste	C Brumley—DPIRD	2018
O1_2	*Lepisiota frauenfeldi*	Adult	Oman	Intercept—DPIRD	2003
O1_3	*Lepisiota frauenfeldi*	Adult	Oman	Intercept—DPIRD	2003
I1_3	*Lepisiota frauenfeldi*	Adult	Kaskan	A Szito—DPIRD	2016
T1_1	*Lepisiota frauenfeldi*	Adult	Kewdale, WA, Australia	Marc Widmer	2017
T1_2	*Lepisiota frauenfeldi*	Adult	Kewdale, WA, Australia	Marc Widmer	2017
T1_3	*Lepisiota frauenfeldi*	Adult	Kewdale, WA, Australia	Marc Widmer	2017
T2_3	*Lepisiota frauenfeldi*	Adult	Kewdale, WA, Australia	Marc Widmer	2017
W1_1	*Lepisiota frauenfeldi*	Adult	Welshpool, WA, Australia	Marc Widmer	2016
W1_2_3	*Lepisiota frauenfeldi*	Pupa	Welshpool, WA, Australia	Marc Widmer	2016
W1_2p_1	*Lepisiota frauenfeldi*	Pupa	Welshpool, WA, Australia	Marc Widmer	2016
W1_2p_2	*Lepisiota frauenfeldi*	Pupa	Welshpool, WA, Australia	Marc Widmer	2016
W1_2p_3	*Lepisiota frauenfeldi*	Pupa	Welshpool, WA, Australia	Marc Widmer	2016
W1_2p_5	*Lepisiota frauenfeldi*	Pupa	Welshpool, WA, Australia	Marc Widmer	2016
W1_2p_6	*Lepisiota frauenfeldi*	Pupa	Welshpool, WA, Australia	Marc Widmer	2016
W2_1	*Lepisiota frauenfeldi*	Adult	Welshpool, WA, Australia	Marc Widmer	2016
W2_2	*Lepisiota frauenfeldi*	Adult	Welshpool, WA, Australia	Marc Widmer	2016
NF1_1	*Lepisiota frauenfeldi*	Adult	North Fremantle, WA, Australia	Marc Widmer	2020
NF1_2	*Lepisiota frauenfeldi*	Adult	North Fremantle, WA, Australia	Marc Widmer	2020
NF1_3	*Lepisiota frauenfeldi*	Adult	North Fremantle, WA, Australia	Marc Widmer	2020
NT1_1	*Lepisiota frauenfeldi*	Adult	NT, Australia	NT Govt	2015
NT1_2	*Lepisiota frauenfeldi*	Adult	NT, Australia	NT Govt	2015
Md1_1	*Lepisiota incisa*	Adult	Maddington, WA, Australia	Marc Widmer	2020
Bw1_1	*Lepisiota frauenfeldi*	Adult	Bayswater, WA, Australia	Marc Widmer	2019
Bw1_2	*Lepisiota frauenfeldi*	Adult	Bayswater, WA, Australia	Marc Widmer	2019
Bw2_1	*Lepisiota frauenfeldi*	Adult	Bayswater, WA, Australia	Marc Widmer	2019
BW2_2	*Lepisiota frauenfeldi*	Adult	Bayswater, WA, Australia	Marc Widmer	2019
W3_1	*Lepisiota incisa*	Adult	Welshpool, WA, Australia	Marc Widmer	2020
W3_2	*Lepisiota incisa*	Adult	Welshpool, WA, Australia	Marc Widmer	2020
Rk1_1	*Lepisiota frauenfeldi*	Adult	Rockingham, WA, Australia	Marc Widmer	2020
Rk1_2	*Lepisiota frauenfeldi*	Adult	Rockingham, WA, Australia	Marc Widmer	2020
SI_H	*Lepisiota frauenfeldi*	Adult	Honolulu, Hawaii, USA	B Hoffmann—CSIRO	2019
W4_1	*Lepisiota frauenfeldi*	Adult	Welshpool, WA, Australia	Marc Widmer	2019
W4_2	*Lepisiota frauenfeldi*	Adult	Welshpool, WA, Australia	Marc Widmer	2019
W4_3	*Lepisiota frauenfeldi*	Adult	Welshpool, WA, Australia	Marc Widmer	2019
W5_1	*Lepisiota frauenfeldi*	Adult	Kewdale, WA, Australia	Marc Widmer	2017
W5_2	*Lepisiota frauenfeldi*	Adult	Kewdale, WA, Australia	Marc Widmer	2017
W5_3	*Lepisiota frauenfeldi*	Adult	Kewdale, WA, Australia	Marc Widmer	2017

**Table 2:** Browsing ant mitochondrial genome assembly quality statistics.

Assembly	# Contigs	Largest contig (bp)	Total length (bp)	Reference length (bp)	GC (%)	Reference GC (%)	N50	NG50	L50	LG50	# Total reads	Reference mapped (%)	Reference avg. coverage depth
B1_5	1	17,090	17,090	17,090	18.89	18.88	17,090	17,090	1	1	586,614	0.23	25
B2_4	1	17,430	17,430	17,090	18.82	18.88	17,430	17,430	1	1	5785	1.09	8.1
B2_5	1	17,106	17,106	17,090	18.87	18.88	17,106	17,106	1	1	2,173,702	0.38	287
Br1_2	1	17,149	17,149	17,090	18.72	18.88	17,149	17,149	1	1	572,617	0.38	79
Br1_4	1	17,090	17,090	17,090	18.84	18.88	17,090	17,090	1	1	11,995	0.8	104.9
Br1_7	1	17,114	17,114	17,090	18.78	18.88	17,114	17,114	1	1	749,198	0.51	150
Br2_1	1	17,090	17,090	17,090	18.85	18.88	17,090	17,090	1	1	2,065,859	0.66	258
Br2_4	1	17,120	17,120	17,090	18.85	18.88	17,120	17,120	1	1	2,187,723	0.04	36
Bw1_1	1	17,112	17,112	17,090	18.87	18.88	17,112	17,112	1	1	2,838,882	0.5	839
Bw1_2	1	17,346	17,346	17,090	18.87	18.88	17,346	17,346	1	1	1,760,306	0.46	549
Bw2_1	1	17,164	17,164	17,090	18.87	18.88	17,164	17,164	1	1	1,238,919	0.63	490
BW2_2	1	17,117	17,117	17,090	18.86	18.88	17,117	17,117	1	1	637,361	0.67	287
I1_3	1	17,339	17,339	17,090	18.64	18.88	17,339	17,339	1	1	198,030	1.27	46
Md1_1	1	16,752	16,752	17,090	19.23	18.88	16,752	16,752	1	1	524,897	0.21	44
NF1_1	1	17,090	17,090	17,090	18.86	18.88	17,090	17,090	1	1	5,807,728	0.57	1152
NF1_2	1	17,222	17,222	17,090	18.75	18.88	17,222	17,222	1	1	5,879,940	0.41	1556
NF1_3	1	17,156	17,156	17,090	18.76	18.88	17,156	17,156	1	1	2,435,285	0.6	922
NT1_1	1	17,090	17,090	17,090	18.87	18.88	17,090	17,090	1	1	378,304	0.52	32
NT1_2	1	17,126	17,126	17,090	18.81	18.88	17,126	17,126	1	1	483,902	0.5	64
O1_2	1	17,222	17,222	17,090	18.8	18.88	17,222	17,222	1	1	8895	1.09	44.5
O1_3	1	17,090	17,090	17,090	18.85	18.88	17,090	17,090	1	1	227,255	0.56	28
PA1_6	1	17,116	17,116	17,090	18.85	18.88	17,116	17,116	1	1	403,286	0.49	27
PA1_9	1	17,098	17,098	17,090	18.69	18.88	17,098	17,098	1	1	275,552	0.8	33
PA2_3	1	17,386	17,386	17,090	18.81	18.88	17,386	17,386	1	1	48,954	2.27	35.3
PA2_5	1	17,090	17,090	17,090	18.89	18.88	17,090	17,090	1	1	2,214,177	0.66	405
Rk1_1	1	17,112	17,112	17,090	18.86	18.88	17,112	17,112	1	1	1,263,275	0.7	315
Rk1_2	1	17,113	17,113	17,090	18.87	18.88	17,113	17,113	1	1	7,494,115	0.47	1785
SI_H	1	17,129	17,129	17,090	18.79	18.88	17,129	17,129	1	1	2,375,332	0.32	195
T1_1	1	17,112	17,112	17,090	18.87	18.88	17,112	17,112	1	1	989,144	0.66	180
T1_2	1	17,144	17,144	17,090	18.88	18.88	17,144	17,144	1	1	135,821	0.48	10
T1_3	1	17,129	17,129	17,090	18.85	18.88	17,129	17,129	1	1	1,712,134	0.62	466
T2_3	1	17,274	17,274	17,090	18.83	18.88	17,274	17,274	1	1	46,526	0.52	9.2
TL1	1	17,090	17,090	17,090	18.85	18.88	17,090	17,090	1	1	240,608	1.97	165
TL2	1	17,111	17,111	17,090	18.75	18.88	17,111	17,111	1	1	20,329	1.08	10
W1_1	1	17,116	17,116	17,090	18.87	18.88	17,116	17,116	1	1	1,223,499	0.58	371
W1_2_3	1	17,223	17,223	17,090	18.86	18.88	17,223	17,223	1	1	1,671,185	0.25	191
W1_2p_1	1	17,132	17,132	17,090	18.7	18.88	17,132	17,132	1	1	28,906	0.15	18.2
W1_2p_2	1	17,107	17,107	17,090	18.86	18.88	17,107	17,107	1	1	3,746,307	0.31	175
W1_2p_3	1	17,111	17,111	17,090	18.84	18.88	17,111	17,111	1	1	27,075	0.97	87.9
W1_2p_5	1	17,181	17,181	17,090	18.79	18.88	17,181	17,181	1	1	15,819	0.53	40
W1_2p_6	1	17,116	17,116	17,090	18.86	18.88	17,116	17,116	1	1	1,196,825	0.44	85
W2_1	1	17,105	17,105	17,090	18.87	18.88	17,105	17,105	1	1	251,384	2.37	186
W2_2	1	17,106	17,106	17,090	18.86	18.88	17,106	17,106	1	1	142,607	1.1	47
W3_1	1	16,478	16,478	17,090	19.07	18.88	16,478	16,478	1	1	2,059,926	0.56	840
W3_2	1	16,455	16,455	17,090	19.19	18.88	16,455	16,455	1	1	3,846,684	0.48	1261
W4_1	1	17,111	17,111	17,090	18.87	18.88	17,111	17,111	1	1	855,920	0.41	166
W4_2	1	17,107	17,107	17,090	18.88	18.88	17,107	17,107	1	1	1,856,809	0.3	307
W4_3	1	17,284	17,284	17,090	18.86	18.88	17,284	17,284	1	1	989,304	0.52	268
W5_1	1	17,115	17,115	17,090	18.86	18.88	17,115	17,115	1	1	370,556	4.26	424
W5_2	1	17,116	17,116	17,090	18.85	18.88	17,116	17,116	1	1	61,510	4.57	66
W5_3	1	17,111	17,111	17,090	18.86	18.88	17,111	17,111	1	1	1,597,430	0.88	447
Minimum	1	16,455	16,455	17,090	18.64	18.88	16,455	16,455	1	1	5785	0.04	8
Maximum	1	17,430	17,430	17,090	19.23	18.88	17,430	17,430	1	1	7,494,115	4.57	1785
Average	1	17,116	17,116	17,090	18.85	18.88	17,116	17,116	1	1	1,332,043	0.82	306
Median	1	17,114	17,114	17,090	18.86	18.88	17,114	17,114	1	1	749,198	0.56	166

The phylogenetic analysis data set contained whole mitogenome sequences of three replicates of the outgroup species (*Lepisiota incisa*) (Md1_1, W3_1 and W3_2), four from the Brisbane incursions, two from Northern Territory, 34 from Western Australia representing 10 browsing ant incursions across Australia (Table 1). The data set also included six browsing ants from overseas; one from Iran, two from Oman, two from Timor and one from Hawaii (Table 1).

**Figure 1:**
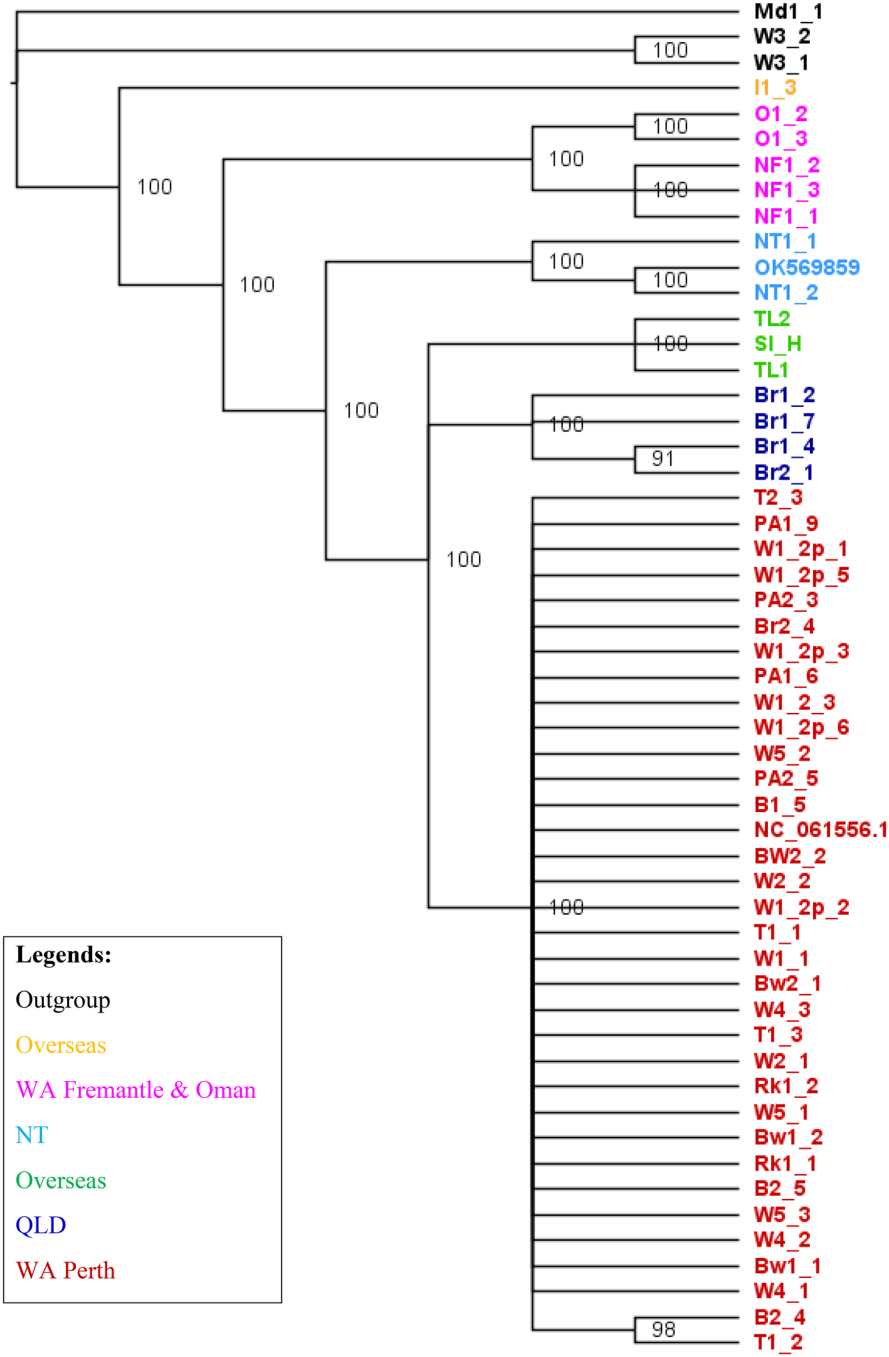
The Bayesian phylogenetic tree of the browsing ants inferred from mitochondrial genome sequence data. The tree was built using the MrBayes v3.2^56^ program that implemented the ‘invgamma’ model and visualised using the FigTree v1.4.4^64^ software. The numbers on the nodes represent the posterior probability percentages. The taxa are coloured to indicate potentially separate incursions. Red: Perth, WA; Pink: Fremantle, WA, and Oman; Dark blue: QLD; Light blue: NT; Light green: Timor Leste and Hawaii; Yellow: Iran; Black: outgroup (*L. incisa*). Taxa NC_061556.1 and OK569859 are retrieved from Tay et al. 2022^55^.

**Figure 2:**
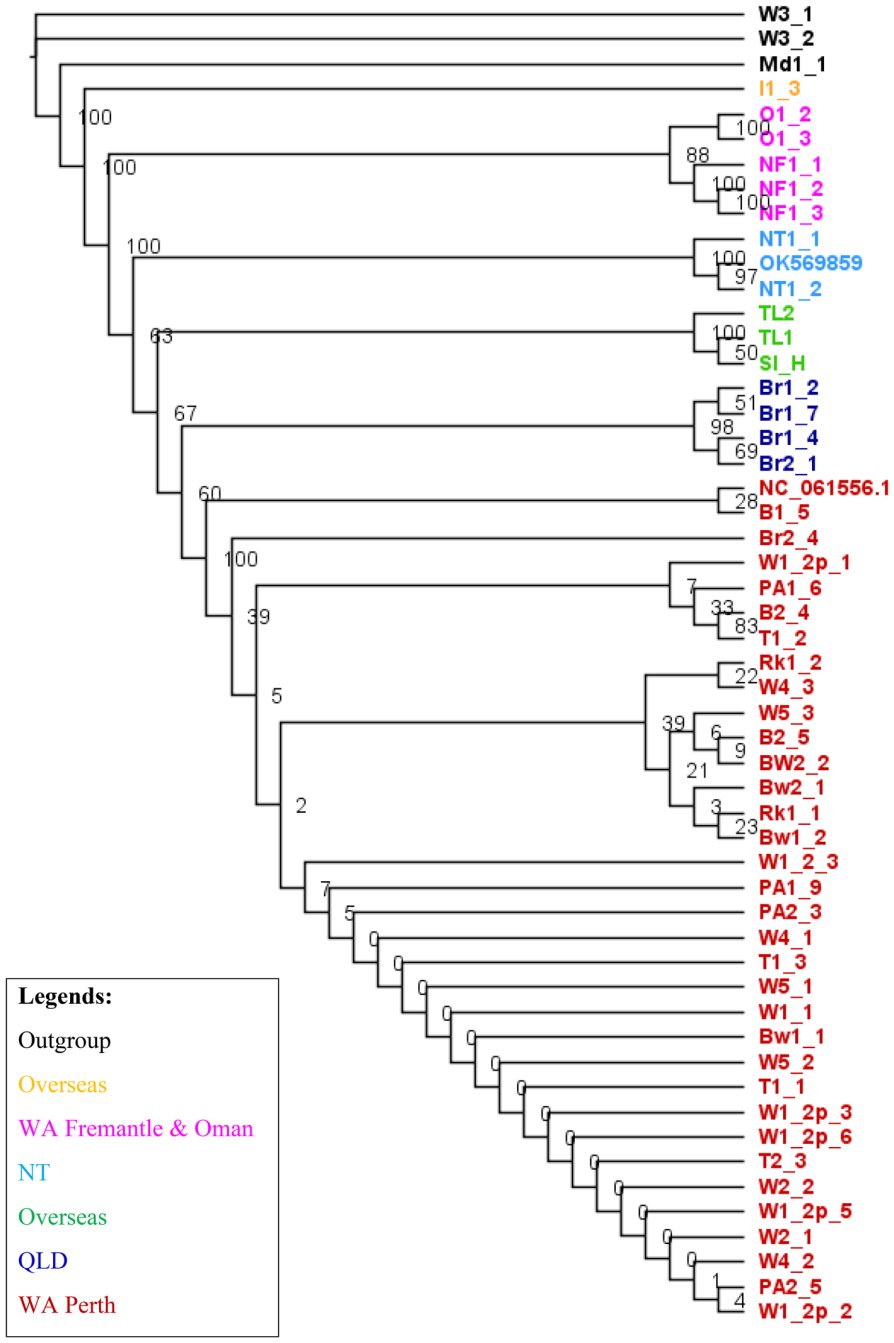
The maximum-likelihood (ML) phylogenetic tree of the browsing ants inferred from mitochondrial genome sequence data. The tree was built using the RAxML v8^58^ (Randomized Axelerated Maximum Likelihood) program that implemented the ‘GTRGAMMA’ model, and visualised using the FigTree^64^ software. The numbers on the nodes represent the percentages of 1000 bootstrap values. The taxa are coloured to indicate potentially separate incursions. Red: Perth, WA; Pink: Fremantle, WA, and Oman; Dark blue: QLD; Light blue: NT; Light green: Timor Leste and Hawaii; Yellow: Iran; Black: outgroup (*L. incisa*). Taxa NC_061556.1 and OK569859 are retrieved from Tay et al. 2022^55^.

We constructed the phylogenetic tree using Bayesian and Maximum-Likelihood methods. Both methods recovered a similar topology (Figs. 1 and 2). The replicates from the same browsing ant incursions clustered together with strong posterior probability support ranging from 90 to 100 (Fig. 1) except for Br2_4. Br2_4 is a Brisbane incursion but grouped with Western Australian incursions in both Bayesian and Maximum-Likelihood based phylogenetic tress (Figs. 1 and 2). The outgroup species formed distinct clades (Figs. 1 and 2). Both methods revealed the incursions from Western Australia as two distinct taxa represented by red and pink colours (Figs. 1 and 2). The pink incursion fell as a sister taxon to the browsing ants from Oman with a support of 100% posterior probability (Fig. 1) and 88% bootstrapping (Fig. 2). The incursions from Northern Territory and Brisbane except Br2_4 appeared as separate clades. The incursions from Timor Leste and Hawaii grouped in one clade (Figs. 1 and 2). The browsing ants from Iran formed an individual clade on both trees (Figs. 1 and 2).

### Mitogenome annotation

**Figure 3:**
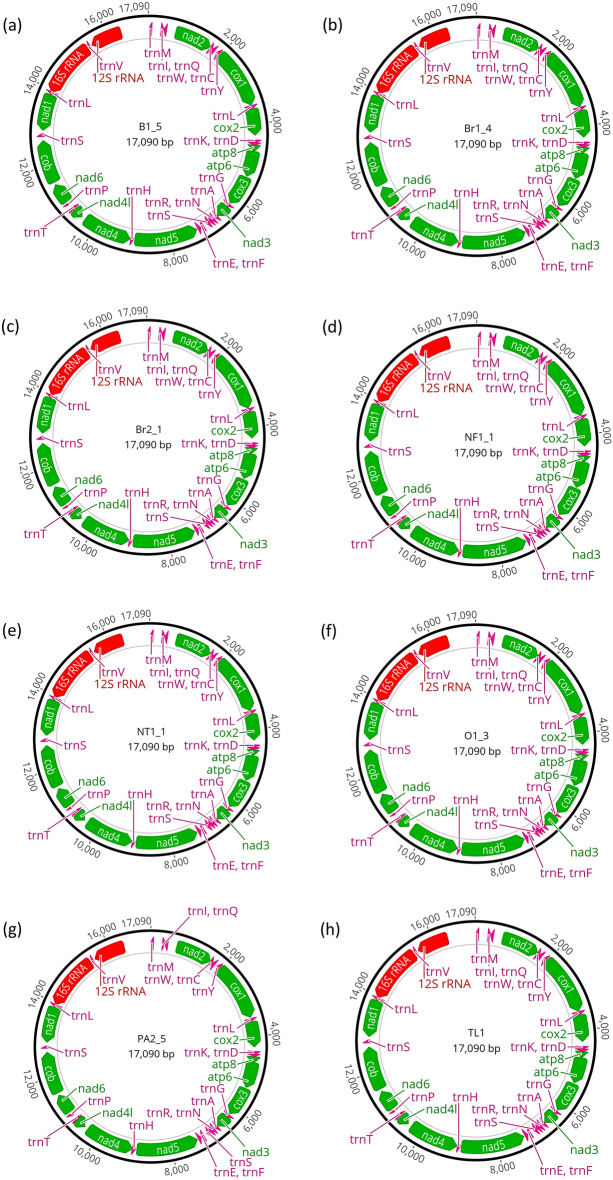
The mitochondrial genomes of eight *Lepisiota frauenfeldi* individuals collected from different locations. (**a**) specimen B1_5, collected from Belmont, Western Australia, Australia; (**b**) specimen Br1_4, collected from Brisbane, Queensland, Australia; (**c**) specimen Br2_1, collected from Brisbane, Queensland, Australia; (**d**) specimen NF1-1, collected from North Fremantle, Western Australia, Australia; (**e**) specimen NT1-1, collected from Northern Territory, Australia; (**f**) specimen O1-3, collected from Oman; (**g**) specimen PA2_5, collected from Perth Airport, Western Australia, Australia; and (**h**) specimen TL1, collected from Timor Leste. Protein coding genes, ribosomal RNAs (rRNA) and the transfer RNAs (tRNA) are represented by the green, red, and pink arrows, respectively.

We annotated the mitogenomes of eight *Lepisiota frauenfeldi* individuals selected from the distinct clades of the Bayesian phylogenetic tree (Fig. 1). The *Lepisiota frauenfeldi* mitogenomes were a typical circular molecule with 17,090 base pairs (bp) in size (Fig. 3a–h) and consisted of 13 protein-coding genes (PCGs), 22 tRNA genes and two rRNA genes (Fig. 3a–h).

### Bayesian phylogenetic tree inferred from a single mitochondrial protein coding gene

**Figure 4:**
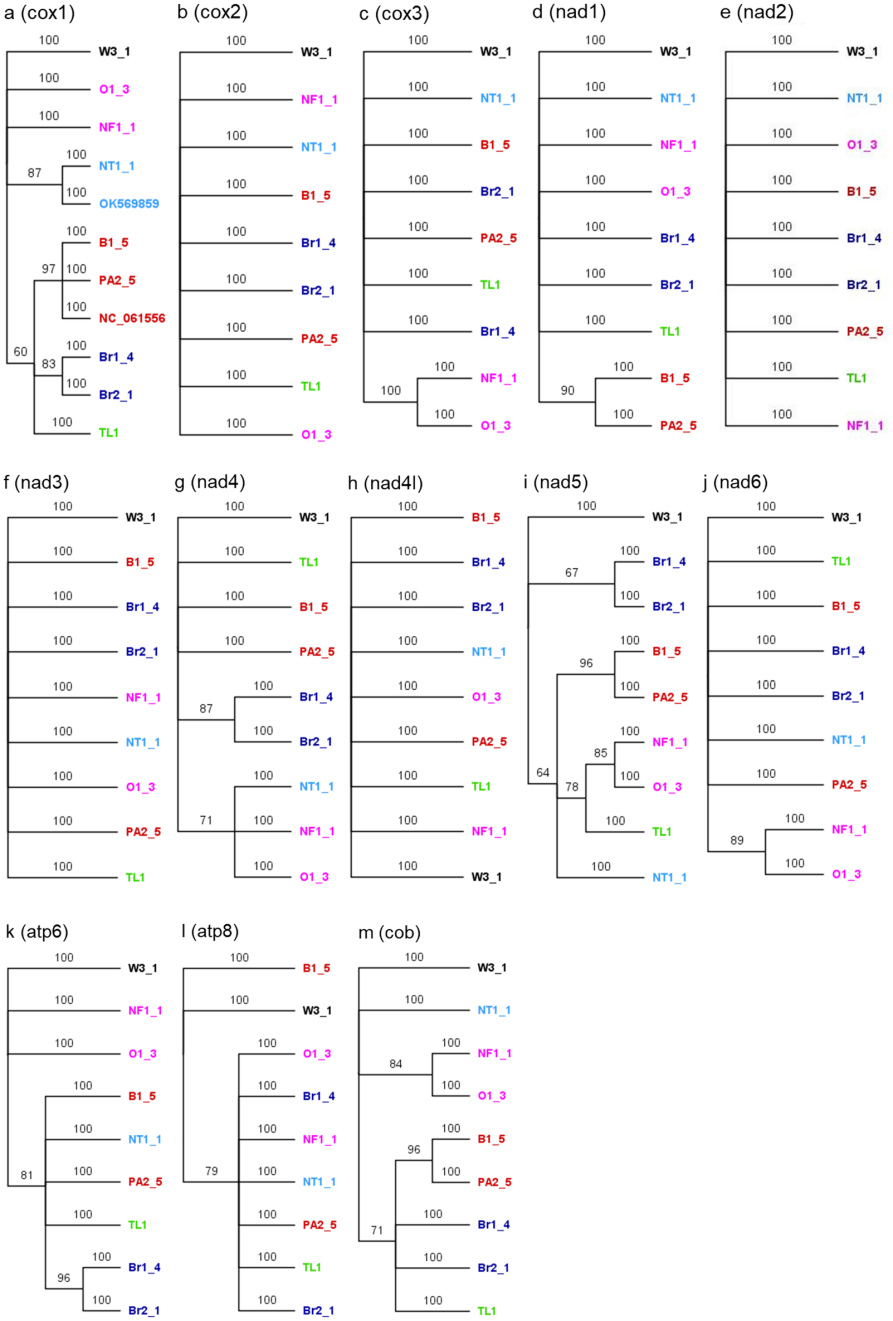
The Bayesian phylogenetic tree of the browsing ants inferred from a single mitochondrial protein coding gene (PCG) sequence data. The tree was built using the MrBayes v3.2^56^ program that implemented the ‘invgamma’ model and visualised using the FigTree v1.4.4^64^ software. The numbers on the nodes represent the posterior probability percentages. The tip labels are coloured to indicate that the same-coloured tips are grouped together in a clade when using the whole mitochondrial genome sequences for Bayesian phylogeny (Fig. 1). Red: Perth, WA; Pink: Fremantle, WA, and Oman; Dark blue: QLD; Light blue: NT; Light green: Timor Leste; Black: outgroup (*L. incisa*).

We extracted 13 PCGs that were present in all the annotated mitogenomes and carried out Bayesian phylogenetic analysis using a single PCG (Fig. 4). Phylogenetic tree using *cox1* gene (Fig. 4a) showed the outgroup (W3-1), Oman (O1-2), and North Fremantle (NF1-1) (Western Australia) incursions on distinctly different branches, instead of clustering Oman (O1-2) and North Fremantle (NF1-1) together as in the mitogenome-based tree (Fig. 1). *cox2* gene-based tree showed that all incursions were separate i.e., no relatedness among the incursions (Fig. 4b). Likewise, all gene-based trees except nad5 showed that even the replicates of the same incursion sites such as Brisbane (Br1_4 and Br2_1) and Western Australia (B1_5 and PA2_5) were different (Fig. 4b–h,j–m). However, nad5 based tree grouped the Northern Territory (NT1-1) incursion together with Western Australian (NF1_1, B1_5 and PA2-5) and overseas incursions (O1_3 and TL1) (Fig. 4i) starkly contrasting to the topology of the mitogenome based trees (Figs. 1 and 2).

**Figure 5:**
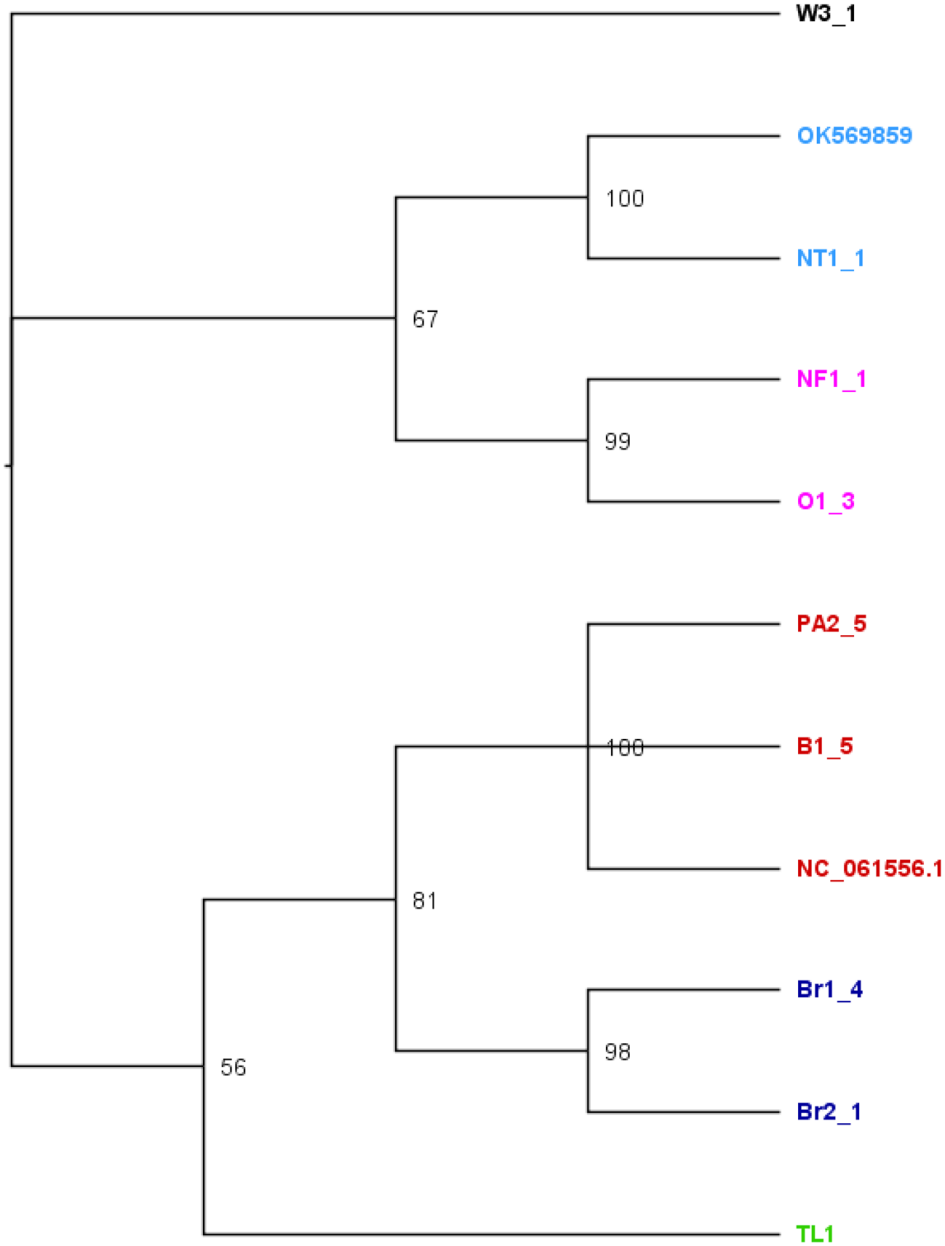
The Bayesian phylogenetic tree of the browsing ants inferred from the whole mitochondrial genome sequences. The tree was built using the MrBayes v3.2^56^ program that implemented the ‘invgamma’ model and visualised using the FigTree v1.4.4^64^ software. The numbers on the nodes represent the posterior probability percentages. The tip labels are coloured to indicate that the same-coloured tips are grouped together in a clade when using the whole mitochondrial genome sequences for Bayesian phylogeny (Fig. 1). Red: Perth, WA; Pink: Fremantle, WA, and Oman; Dark blue: QLD; Light blue: NT; Light green: Timor Leste; Black: outgroup (*L. incisa*). Taxa NC_061556.1 and OK569859 are retrieved from Tay et al. 2022^55^.

However, when the whole mitogenome sequences were used instead of just a single PCG, the Bayesian phylogenetic tree (Fig. 5) revealed the same topology as in Fig. 1. The relatedness among the incursions was revealed with a greater resolution, and 100% posterior probability support on the tree branches and 50–100% on the clades (Fig. 5). This mitogenome based tree separated the outgroup from all the other sequences, clustered the replicates of the same incursion together, and revealed that the browsing ant incursions in Western Australia (B1-5, PA2-5 and NF1-1), Northern Territory (NT1-1), Brisbane (Br1-4 and Br2-1) and Timor Leste (TL1) were all separate (Fig. 5). Furthermore, it showed that Western Australia had two separate incursions (B1-5, PA2-5 and NF1-1). Interestingly, the mitogenome based tree topology showed independence of sample sizes (Figs. 1 and 5), for example, 51 mitogenomes in Fig. 1 and nine mitogenomes in Fig. 5, but the topology demonstrated the same resolution.

### Mitogenome wide nucleotide identity

**Figure 6:**
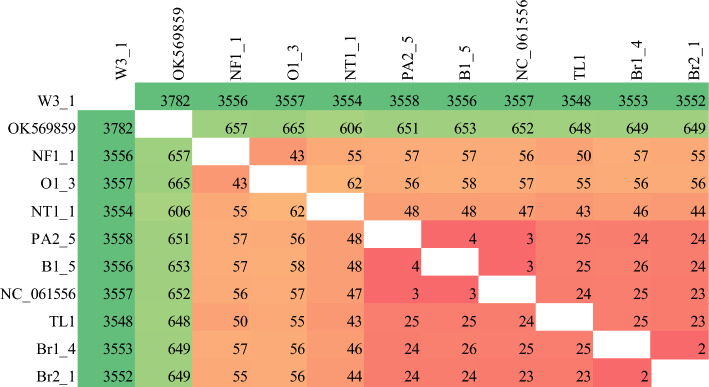
Number of bases varying in the Browsing ant mitogenomes (17,090 bases) across different incursions. Taxa NC_061556.1 and OK569859 are retrieved from Tay et al. 2022^55^.

The nucleotide identity matrix extracted from the sequence alignment showed that *L. frauenfeldi* and *L. incisa* (outgroup) mitogenomes had about 20% (about 3500 bases out of 17,000) nucleotide variation (Fig. 6). The *L. frauenfeldi* mitogenomes varied between each other by only 2–650 bases (Fig. 6). However, none of the *L. frauenfeldi* mitogenomes had 100% nucleotide identity when compared to each other (Fig. 6).

### Individual gene wide nucleotide identity

**Figure 7:**
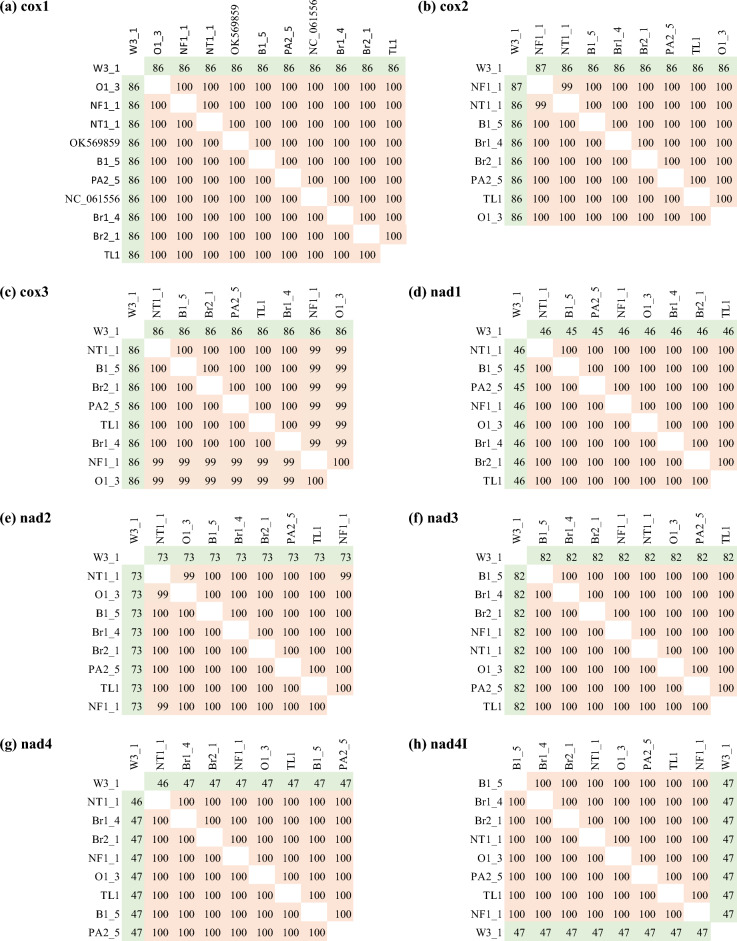

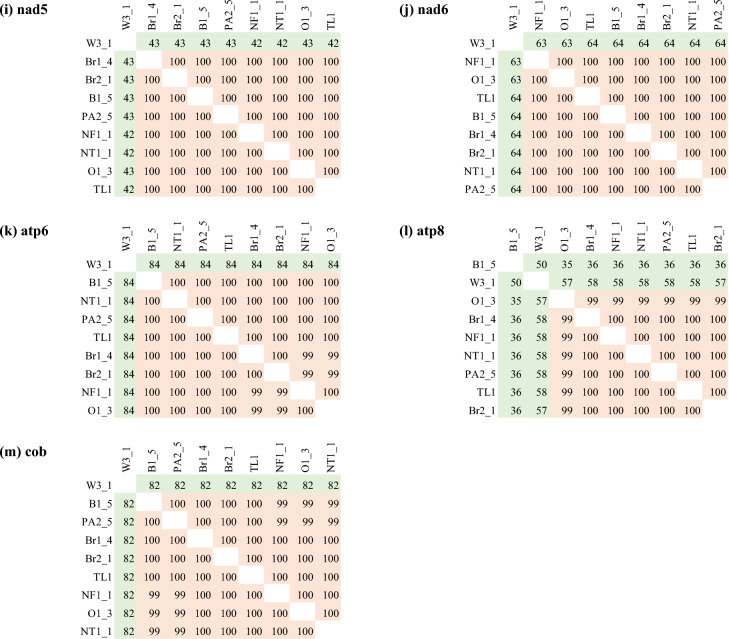
Distance matrix of nucleotide alignment for individual Protein Coding Genes (PCGs). These alignments were used for constructing individual gene-wise Bayesian phylogenetic trees (Fig. 5).

We extracted the nucleotide identity matrix from the individual gene sequence alignments that were used for constructing the Bayesian trees for each of the PCGs (Fig. 4). The matrices showed that there was always 100% nucleotide identity between some pairs of browsing ant individuals when considered only a single gene (Fig. 7).

## Discussion

We sequenced and analysed the whole mitogenome to determine the relatedness of the browsing ant incursions from Australia and overseas.

Bayesian inference based phylogenetic analysis using the whole mitogenome sequences showed that Western Australian incursions were grouped into two clusters, one was in Fremantle (Fig. 1; pink cluster) and the other was across the rest of the state (Fig. 1; red cluster). The incursions within the red cluster might be a spread of a single incursion and/or might be multiple incursions from the same source/origin (Fig. 1; red cluster). It is interesting to note that one sample from Brisbane (Br2-4) also grouped with the main Western Australian cluster (red in colour), indicating a possible spread, or multiple inductions to Australia from a common origin. Furthermore, Western Australian clusters were separate from those in other Australian states and overseas with more than 90% posterior probability support on the clades (Fig. 1). Maximum-Likelihood method inferred the same topology (Fig. 2).

We further analysed the mitogenomes to determine if a single gene based phylogenetic tree can inform the relatedness of the incursions with a similar resolution like that with the whole mitogenomes. A single gene based phylogenetic tree will allow for developing a fast Polymerase Chain Reaction (PCR) based tool to diagnose future browsing ant incursions without needing to sequence the entire genome^46^. For this purpose, we annotated a subset of *L. frauenfeldi* mitogenomes comprising at least a representative from each distinct clade of the phylogenetic tree (Fig. 1). Annotation of the mitogenomes showed that all 13 typical protein coding genes (PCGs) in insect mitogenomes^47–49^ were present in *L. frauenfeldi* (Fig. 3). We also extracted the PCG sequences from one of our *L. incisa* samples to be included as outgroup in the gene-based phylogenetic analysis.

We continued our analysis with 13 PCGs and performed Bayesian phylogenetic analysis using a single PCG at a time. Our results demonstrated that none of the 13 PCGs could reveal the relatedness among the incursions unlike the mitogenome based trees (Figs. 1, 2 and 5). Our findings agree with the recent studies that the whole mitogenomes reveal phylogenetic relationships at a deeper level compared to those inferred from a single mitochondrial gene^41,43,44,50,51^. However, a single mitochondrial gene such as *cox1* might still be an effective tool for barcoding animal species for species ID, though not likely to reveal effective relatedness within that species^52–54^.

The nucleotide identity analysis (Fig. 6) of our subset of mitogenomes offered an explanation as to why a single mitochondrial gene could not reveal the relatedness of the browsing ant incursions. This was because there were only 2- 62 base variations across the entire *L. frauenfeldi* mitogenomes except the OK569859 mitogenome (Fig. 6). OK569859 mitogenome was retrieved from NCBI^55^. It showed a 606–665 base variation when compared to the other mitogenomes. This high nucleotide variation accounts for the fact that the OK569859 mitogenome sequence itself has 141 N’s, and in alignment with most of the other genomes in Fig. 6, it has several big gaps. However, these gaps and missing columns in the alignment are ignored for the tree construction purposes in both Bayesian^56,57^ and Maximum-Likelihood^58^ algorithms as these algorithms use nucleotide substitution-based models. What this all boils down to is that there is a limited phylogenetic signal in the *L. frauenfeldi* mitogenome. These signals are further reduced in any fragment of the *L. frauenfeldi* mitogenome as demonstrated by the 100% nucleotide identity between genes in the nucleotide identity matrices (Fig. 7). The 100% nucleotide identity between any pair of genes meant that the corresponding gene had no phylogenetic signal for that pair of individuals (i.e., incursions) to separate them in the tree. And there was always 100% nucleotide identity between some pairs of individuals in any of the 13 PCGs (Fig. 7).

Tay et al. 2022^55^ is an example of how a single mitochondrial gene can be limited for determining the relatedness of the browsing ant incursions. The study carried out a phylogenetic analysis of browsing ants using a partial *cox1* gene (546 bp)^55^. The authors have claimed that the Brisbane and Perth browsing ant incursions might originate from India (Jodhpur) and Pakistan (Punjab), respectively, because of sharing 100% *cox1* sequence. The maximum likelihood based phylogenetic tree has clustered them on the same clade with more than 90% bootstrap support^55^. However, our analysis based on the whole mitogenome shows that this may not be the case. While there are more *cox1* genes than full mitogenomes available from NCBI, we have demonstrated here that this may not be enough information to accurately determine the relatedness between incursions. As well as using the partial *cox1,* Tay et al., 2022 did submit their draft mitogenomes of Perth (NC_061556.1) and Brisbane (OK569859) browsing ants^55^ to NCBI^45^ and we retrieved them and included in our phylogenetic analysis (Figs. 1, 2 and 5). The mitogenome based tree clustered Perth and Brisbane browsing ants on distinctly different clades (Figs. 1, 2 and 5). Based on this analysis, we can confidently conclude that the Brisbane and Perth browsing ant incursions are separate introductions. This is in contrast to the resolution obtained by partial *cox1* gene analysis^55^.

The entirety of our results demonstrated that whole mitogenome based phylogenetic analysis was warranted to determine the phylogenetic relationship among the incursions. Using an individual PCG including the widely used *cox1* could not reveal the relatedness of the browsing ant incursions in Australia.

Furthermore, to the best of our knowledge, our results provide a world first draft mitogenome of *L. incisa*, a closely related species to *L. frauenfeldi*, therefore a suitable outgroup for this browsing ant phylogenetic analysis. Taken together, our contribution of 48 *L. frauenfeldi* and three *L. incisa* mitogenomes will serve as an excellent resource for those wanting to trace any future browsing ant infestations in Australia. This will allow us to determine the relatedness of future browsing ant incursions. In other words, we will be able to address the biosecurity questions like “are the new browsing ant infestations in Australia a spread of the existing incursions, or separate introductions from the same or separate origins, or a combination of both?”. For this biosecurity surveillance purpose, our method i.e., whole mitochondrial genome-based phylogenetic analysis has proven to be affordable, scalable, and effective. Others who wish to pursue further into the population level analysis may wish to do so using the STR or microsatellite markers.

The information on the relatedness of browsing ant incursions in Australia is crucial in evaluating the risk of finding further infestations of browsing ant across Australia, and also in assessing the continued risk of future incursion. Currently, all known infestations in Australia are under eradication, and we expect that this process will be successful. Our findings also lay the foundation for further research to determine where from overseas the separate browsing ant incursions have hailed from. If we can determine the source of our incursions, we will stand better placed to implement mitigation procedures along the high-risk pathways, and also border and post-border biosecurity measures at home to reduce our risk.

## Materials and methods

### Materials

Browsing ants were collected from various incursions in Western Australia, Queensland and overseas complying with all relevant institutional, national, and international guidelines and legislation. Fifty-one individuals from these incursions were used for mitochondrial genome sequencing (Table 1).

### DNA extraction and sequencing library preparation

Total DNA was extracted from a leg biopsy of the collected Browsing ants using Qiagen’s DNeasy Blood & Tissue Kits (Hilden, Germany) following the manufacturer’s instructions. The extracted DNA was quantified using a Qubit 2.0 Fluorometer (Thermo Fisher Scientific). DNA library for MinION sequencing was prepared using Oxford Nanopore Technologies’ PCR barcoding genomic DNA (SQK-LSK109) kit following the manufacturer’s instructions. The library was quantified using a Qubit 2.0 Fluorometer.

### Loading the library for sequencing

A 75 µL loading reaction was made as follows: 37.5 μL Sequencing Buffer (SQB), 25.5 μL Loading Beads (LB) and 12 μL DNA library. A priming mixture was made by adding 30 μl of thawed and mixed Flush Tether (FLT) directly into a new thawed and mixed Flush Buffer (FB) tube. Then, the library reaction and the priming mix were loaded into the flow cell (FLO‐MIN106D, R9.5) of the MinION sequencer as per the manufacturer’s instructions.

### Mitochondrial genome assembly

**Figure 8:**
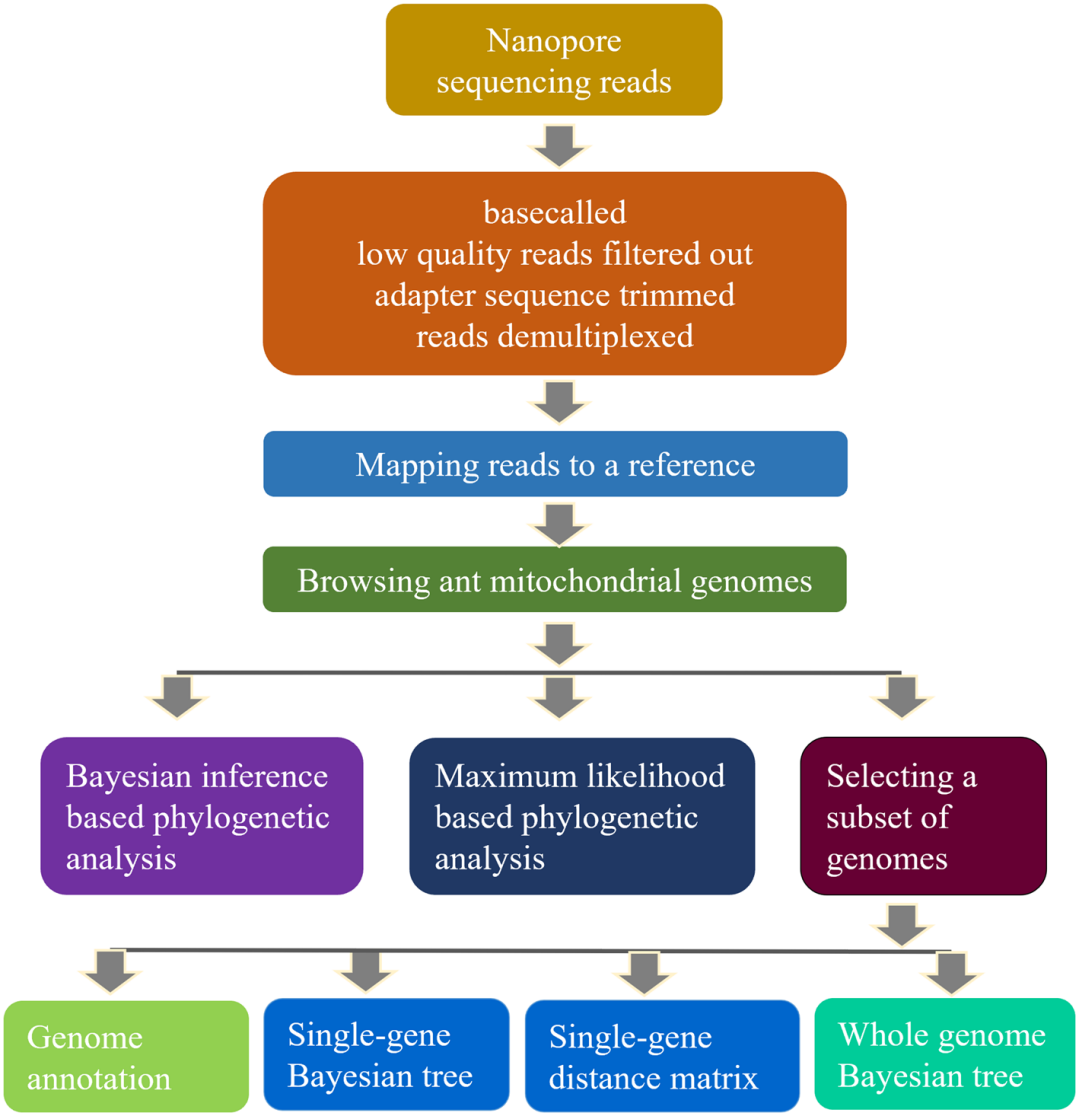
Computational workflow developed and implemented for determining the relatedness of the browsing ant incursions in Australia. *BLAST* basic local alignment search tool, *ANI*, average nucleotide identity.

The nanopore electrical signals were basecalled using the guppy (version 6.0.1 + 652ffd179) (https://community.nanoporetech.com/downloads) ‘high-accuracy’ mode. The reads were trimmed for adapter sequences, demultiplexed and filtered out for low quality reads. Then, the high quality reads were mapped to NC_061556.1, a *Lepisiota frauenfeldi* mitochondrial genome^55^ (Fig. 8). For the low-quality mitochondrial genome assemblies, the nanopore electrical signals were basecalled again using the guppy ‘fast’ mode and the above quality control and mapping steps were repeated. For *L. incisa*, the first version of the mitochondrial genome was generated by mapping the reads of a *L. incisa* sample (W3_1) to *L. frauenfeldi*, NC_061556.1^55^. The W3_1 mitochondrial genome assembly was improved by two rounds of mapping back the W3_1 reads to the W3_1 first-version mitochondrial genome. Then, the draft W3_1 mitochondrial genome was used as a reference to map W3_2 reads using minimap2^59^ (v 2.24-r1122), and assembly Md1_1 reads using miniasm^60^ (v 0.3-r179) as miniasm produced better assembly than minimap2.

### Phylogenetic analysis

The final mitochondrial genomes of 48 *L. frauenfeldi* and three *L. incisa* were used for the phylogenetic analysis. The mitogenomes were aligned using Geneious^61^ and MUSCLE^62^ alignment (v3.8.425) under the default parameters. The alignment was exported out of Geneious and subjected to phylogenetic analysis using both Bayesian inference^56,57^ and Maximum-Likelihood^58^ methods. For Bayesian inference-based tree, the parameters were as follows: lset nst = 6 rates = invgamma; propset ExtTBR$prob = 0; mcmc ngen = 1,000,000 printfreq = 100 samplefreq = 1000 diagnfreq = 1000 nchains = 4 savebrlens = yes; sumt burnin = 12,500; sump burnin = 12,500. For Maximum-Likelihood based tree, the parameters were as follows: raxmlHPC -f a -m GTRGAMMA -p 12,345 −× 12,345 −# 1000.

### Mitochondrial genome annotation

The assembled mitochondrial genome of the browsing ants was annotated using the MITOS^63^ web application (http://mitos.bioinf.uni-leipzig.de/index.py) and *L. frauenfeldi*, NC_061556.1^55^ as a reference. In MITOS, ‘Invertebrate’ genetic code was applied. Thresholds for the MITOS protein search parameters were default (BLAST E-value Exponent = 2, Cutoff = 50, Maximum Overlap = 20, Clipping Factor = 10, Fragment Overlap = 20, Fragment Quality Factor = 10, Start/Stop Range = 6, Final Maximum Overlap = 35). The annotated mitochondrial genomes were circularised in Geneious^61^ using the assembled mitochondrial genome and the GFF (General Feature Format) file containing the annotation information.

### Ethical approval

No ethical approval was required as both *Lepisiota frauenfeldi* and *Lepisiota incisa* are pest species.

## Data Availability

The mitochondrial genomes generated in the study have been deposited in the National Centre for Biotechnology Information (NCBI) and are publicly available at https://www.ncbi.nlm.nih.gov/ under the following GenBank accession numbers: B2_4 (OQ561465), B2_5 (OQ561466), Br1_2 (OQ561467), Br1_7 (OQ561468), Br2_4 (OQ561469), Bw1_1 (OQ561470), Bw1_2 (OQ561471), Bw2_1 (OQ561472), BW2_2 (OQ561473), I1_3 (OQ561474), Md1_1 (OQ561475), NF1_2 (OQ561476), NF1_3 (OQ561477), NT1_2 (OQ561478), O1_2 (OQ561479), PA1_6 (OQ561480), PA1_9 (OQ561481), PA2_3 (OQ561482), Rk1_1 (OQ561483), Rk1_2 (OQ561484), SI_H (OQ561485), T1_1 (OQ561486), T1_2 (OQ561487), T1_3 (OQ561488), T2_3 (OQ561489), TL2 (OQ561490), W1_1 (OQ561491), W1_2_3 (OQ561492), W1_2p_1 (OQ561493), W1_2p_2 (OQ561494), W1_2p_3 (OQ561495), W1_2p_5 (OQ561496), W1_2p_6 (OQ561497), W2_1 (OQ561498), W2_2 (OQ561499), W3_1 (OQ561500), W3_2 (OQ561501), W4_1 (OQ561502), W4_2 (OQ561503), W4_3 (OQ561504), W5_1 (OQ561505), W5_2 (OQ561506), W5_3 (OQ561507), B1_5 (OQ612679), Br1_4 (OQ612680), Br2_1 (OQ612681), NF1_1 (OQ612682), NT1_1 (OQ612683), O1_3 (OQ612684), PA2_5 (OQ612685), TL1 (OQ612686).
